# A161 HEMOPEXIN TREATMENT ALLEVIATES COLITIS SEVERITY IN THE ABSENCE OF IL-22RA1 SIGNALING IN MICE

**DOI:** 10.1093/jcag/gwae059.161

**Published:** 2025-02-10

**Authors:** A S Ajayi, C Gerkins, T Cuisiniere, C McCartney, T Maumy, R Hajjar, M Oliero, G Fragoso, A Calve, M M Santos

**Affiliations:** Centre de Recherche du Centre Hospitalier de l’Universite de Montreal, Montreal, QC, Canada; Centre de Recherche du Centre Hospitalier de l’Universite de Montreal, Montreal, QC, Canada; Centre de Recherche du Centre Hospitalier de l’Universite de Montreal, Montreal, QC, Canada; Centre de Recherche du Centre Hospitalier de l’Universite de Montreal, Montreal, QC, Canada; Centre de Recherche du Centre Hospitalier de l’Universite de Montreal, Montreal, QC, Canada; Centre de Recherche du Centre Hospitalier de l’Universite de Montreal, Montreal, QC, Canada; Centre de Recherche du Centre Hospitalier de l’Universite de Montreal, Montreal, QC, Canada; Centre de Recherche du Centre Hospitalier de l’Universite de Montreal, Montreal, QC, Canada; Centre de Recherche du Centre Hospitalier de l’Universite de Montreal, Montreal, QC, Canada; Centre de Recherche du Centre Hospitalier de l’Universite de Montreal, Montreal, QC, Canada

## Abstract

**Background:**

Inflammatory bowel disease (IBD) is often defined by persistent intestinal epithelial inflammation, bleeding, and pain. It is one of the major risk factors for colorectal cancer (CRC). IBD’s pathogenesis is fundamentally characterized by the release of free luminal heme. Dietary heme induces intestinal dysbiosis and exacerbates colitis. Interleukin 22 (IL-22) is produced in the gut by immune cells, mainly innate lymphoid cell 3 (ILC3). It is known to induce a protective response mediated by hemopexin, a plasma heme scavenger produced in the liver that limits the availability of heme iron to microbes and suppresses bacterial growth.

**Aims:**

To evaluate the protective role of IL-22 by the stimulation of hemopexin in a mouse model of acute colitis

**Methods:**

To assess the effect of IL-22 on colitis, *Il22ra1*^*-/-*^ and wild-type (Wt) mice on a C57BL/6 background were treated with 2.5% dextran sodium sulphate (DSS) in drinking water for 12 days to induce colitis, after which they were sacrificed. To reverse colitis severity with hemopexin, three groups of mice received 2.5% DSS in drinking water for 12 days. The treatment group (*Il22ra1*^*-/-*^) received an 15 mg/kg BW hemopexin injection intraperitoneally on day 7 of the DSS treatment, while the other two groups, Wt and *Il22ra1*^*-/-*^, received the vehicle (PBS).

**Results:**

*Il22ra1* ^*-/-*^ mice had significantly greater weight loss, higher disease activity index (DAI) score and shorter colon length on day 12 (endpoint) compared to the Wt mice. Significant increases in inflammatory markers such as lipocalin-2 (lcn-2), TNF-α and IL-6 were observed in *Il22ra1*^*-/-*^ mice, contributing to their condition. Decreased fecal hemopexin and increased free luminal heme were observed in *Il22ra1*^*-/-*^ mice compared to the Wt mice. Hemopexin administration reversed the disease severity by reducing DAI, inflammatory markers and increasing the colon length. Increased fecal hemopexin and reduced luminal heme were also observed in the *Il22ra1*^*-/-*^ mice.

**Conclusions:**

Hemopexin is present in the gut during colitis. The absence of Il-22ra1 enhances free luminal heme production, reduces hemopexin concentration in the gut, and aggravates colitis in mice. Hemopexin administration reduces free luminal heme in the gut, enhances hemopexin availability and alleviates colitis severity in mice.

**Funding Agencies:**

CIHRUniversite de Montreal, CRCHUM, Institute du Cancer de Montreal

